# Osteochondral autograft transplantation versus autologous bone-cartilage paste grafting for the treatment of knee osteochondritis dissecans

**DOI:** 10.1007/s00264-020-04804-6

**Published:** 2020-09-21

**Authors:** Alessandro Di Martino, Simone Silva, Luca Andriolo, Giulia Merli, Davide Reale, Stefano Zaffagnini, Giuseppe Filardo

**Affiliations:** 1 Clinica Ortopedica e Traumatologica II, IRCCS Istituto Ortopedico Rizzoli, Via Pupilli,1/10, 40136 Bologna, Italy; 2 Applied and Translational Research (ATR) Center, IRCCS Istituto Ortopedico Rizzoli, Bologna, Italy

**Keywords:** Osteochondritis dissecans, OCD, Knee, Osteochondral autologous transplantation, Cartilage, Paste grafting

## Abstract

**Purpose:**

To compare the results of two groups of patients affected by osteochondritis dissecans (OCD) of the knee and treated with either osteochondral autologous transplantation (OAT) or bone-cartilage paste grafting (PG).

**Methods:**

A total of 27 patients affected by OCD lesions of the femoral condyles were included: 15 treated with OAT, 12 with PG, with comparable baseline characteristics (mean age 22.4 ± 7.2 vs. 24.2 ± 8.5 *p* = n.s., mean defect size 2.2 ± 1 cm^2^ vs 2.6 ± 1 cm^2^ *p* = n.s.). Patients were evaluated pre-operatively and at 24 and 84 months post-operatively with the International Knee Documentation Committee (IKDC) subjective and objective scores. Sport activity level was evaluated with the Tegner activity score. Adverse events and failures were also recorded.

**Results:**

The IKDC subjective score improved significantly in both groups. At 24 months, a significant improvement from 53.4 ± 9.1 to 80.8 ± 12.9 (*p* = 0.005) was obtained in the OAT group and from 44.6 ± 11.0 to 71.4 ± 25.3 in the PG group (*p* = 0.008). A further statistically significant increase was observed at 84 months in both groups. No significant differences were found between OAT and PG at both follow-ups. One OAT patient required post-operative knee mobilization under narcosis and two complained of donor site symptoms. More failures were documented in the PG vs OAT group (25% vs 0%; *p* = 0.043).

**Conclusion:**

Both PG and OAT provided overall satisfactory results up to 84 months follow-up. However, while PG presents the advantages of a less invasive approach with lower adverse events, the higher failure rate of PG should be considered when choosing between these two surgical treatment options for restoration of the articular surface in patients affected by knee OCD.

## Introduction

Knee osteochondral lesions are a common pathology in orthopaedic practice, generally presenting significant functional limitations and pain, with a quality-of-life impairment similar to osteoarthritis (OA) patients scheduled for knee replacement [1, 2]. If left untreated, osteochondral defects may actually lead to the development of early degenerative OA changes [3, 4]. An exemplary instance is represented by osteochondritis dissecans (OCD), a pathologic process of the osteochondral unit, with a multi-factorial aetiology involving both biological and mechanical factors [5, 6]. It is characterized by sequestration of subchondral bone, with possible evolution to articular cartilage involvement, and ultimately to the detachment of the entire osteochondral unit. The natural history of knee joints after excision of an OCD loose body has shown high rates of OA development and knee arthroplasty at long-term follow-up [7]. This is particularly detrimental, considering that OCD affects especially a young population with long life expectancy. Therefore, several bone and cartilage reconstruction procedures have been developed in order to restore the entire osteochondral unit and thus prevent the detrimental effects of osteochondral defects on joint homeostasis.

Osteochondral autografts are a common procedure for the treatment of osteochondral defects, and various techniques have been described. One of the most studied is osteochondral autologous transplantation (OAT), consisting of the transplantation of one or more osteochondral plugs from less weight-bearing joint areas. The advantages of this technique are the possibility to fill the defect with mature hyaline cartilage and to treat concurrently both cartilage and subchondral bone [8]. To take advantage of the one-step autologous approach while reducing the risk of donor site morbidity, another described procedure is the bone-cartilage paste grafting technique (PG) [9]. This involves the implantation of a mixture articular cartilage and underlying cancellous bone fragments, stimulating cartilage repair by providing an environment to favour the interaction of bone marrow mesenchymal cells with the combination of articular cartilage matrix and live chondrocytes [10]. Both procedures showed positive results also in small OCD surveys [11, 12]. Nevertheless, there is currently no comparative evidence about the results and complications of these approaches to support the use of one over the other.

The aim of this study was to compare the results of two groups of patients affected by OCD and treated with either OAT or PG, to determine advantages and disadvantages of these procedures to address knee osteochondral lesions.

## Material and methods

### Patient selection

An institutional database that prospectively collects clinical outcomes of patients treated with chondral and osteochondral procedures was used for this study, approved by the Hospital Ethics Committee and Internal Review Board of the Rizzoli Orthopaedic Institute, Bologna, Italy (prot. gen. n. 39667), and informed consent of all patients was obtained. The same surgical indications were considered for both OAT and PG treatments: osteochondral defects graded IV on ICRS (International Cartilage Repair Society) classification or OCD graded III–IV on OCD ICRS classification, respectively, located at the femoral condyles or trochlea, sized between 1 and 5 cm^2^, causing knee symptoms (e.g. pain, swelling or locking), which failed a conservative treatment and were not suitable for refixation. Contraindications for the treatment were OA (grade 3–4 Kellgren-Lawrence scale), multiple or bipolar lesions, untreated misalignment or instability (patients who presented with an axial deviation or an ACL lesion underwent a combined surgical procedure of realignment or ligament reconstruction in the same surgical session) and other general medical conditions (e.g. diabetes, rheumatoid arthritis, neoplastic diseases, immunodeficiency disorders, substance abuse). To compare the two procedures in homogeneous cohorts, isolated procedures to address OCD lesions of the tibiofemoral compartment were selected. More in detail, inclusion criteria for the current retrospective analysis of prospectively collected data were patients of all ages, both male and female, affected by isolated OCD lesion involving knee femoral condyles, treated either with single-plug OAT or PG both with arthroscopic or arthrotomic approach and prospectively followed-up up to a mid-term follow-up. Patients affected by other aetiologies than OCD, with defects of the patellofemoral compartment, or treated with combined surgeries (including realignment procedures), were excluded.

### Surgical procedure and rehabilitation protocol

Both surgical procedures were performed in a single step, by different experienced surgeons from the same orthopaedic division. Patients, under general or spinal anaesthesia, were placed in a supine position, and a thigh tourniquet was applied. An open arthrotomy, a mini-arthrotomy or a standard arthroscopic approach was used, depending on the size and site of the lesion. Any loosened fragment was removed, along with excision from the defect of fibrous tissue and degenerated bone, until viable bleeding bone was reached.

**Fig. 1:**
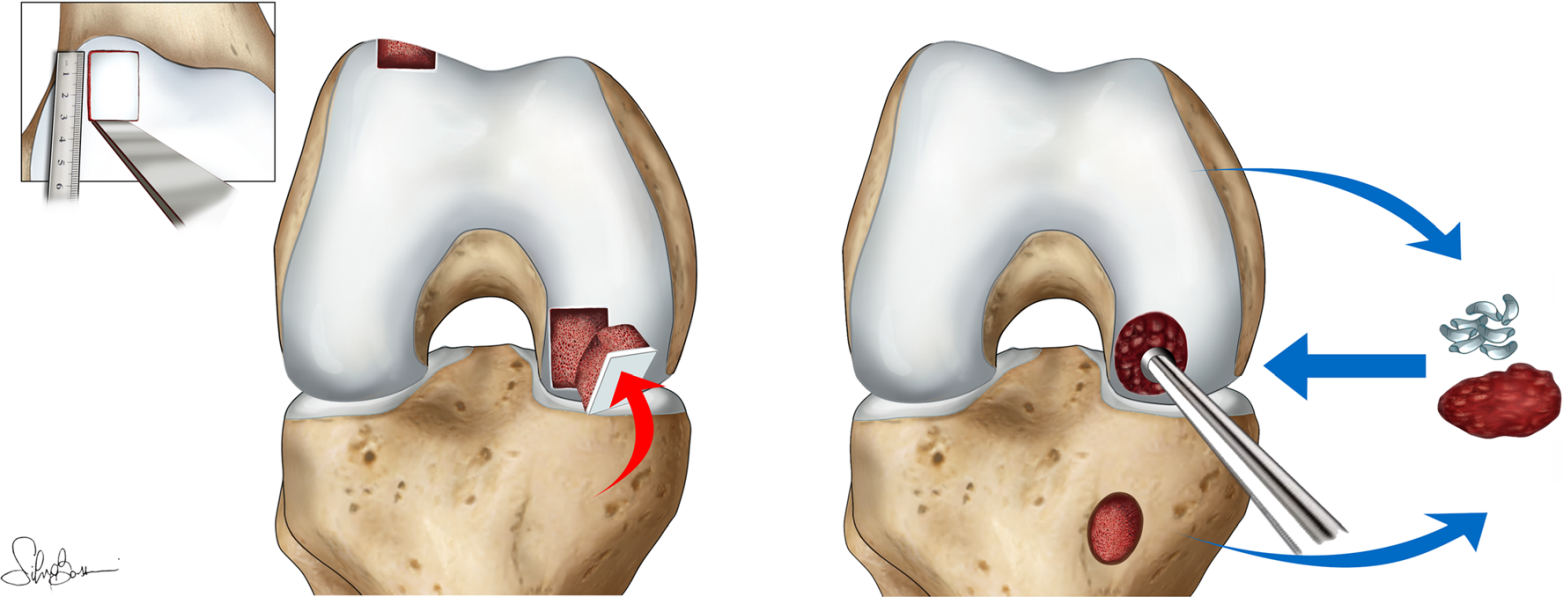
Surgical procedures: left, OAT technique; right, PG procedure

OAT was performed as previously described [13] (Fig. 1). Briefly, it involved the lesion preparation with a chisel to obtain squared margins; the prepared defect was measured, and an osteochondral graft of appropriate size was then removed from a healthy minimal weight-bearing donor zone on the superolateral aspect of the lateral femoral condyle or from the trochlea, preserving the patellar groove. Graft thickness was equal to the recipient site depth so that the graft cartilage surface did not sit either above or below the articular cartilage level of the femoral condyle. The graft bone was carefully contoured so that it fit precisely into the recipient bed. Graft was inserted press-fit unless the stability was judged insufficient, and in such cases, fixation with an absorbable screw was used (seven patients).

PG procedure was performed as previously described [11] (Fig. 1). Briefly, it included multiple penetration of the subchondral bone with an awl until bleeding occurred; cartilage was then harvested from the margin of the intercondylar notch with use of an 8-mm trephine, and cancellous bone was taken from the proximal aspect of the tibia through a mini-incision. Both cartilage and bone were morselized to obtain a paste that was used to cover the osteochondral defect.

Post-operative rehabilitation protocols were comparable for both treatments. Post-operative management focused on early mobilization to facilitate faster resolution of swelling, promote healing and joint nutrition and prevent adhesions. On the second post-operative day, self-assisted mobilization of the knee or continuous passive motion was recommended until 90° of flexion was reached. Patients did not usually require more than two  weeks of continuous passive motion. Early isometric and isotonic exercises were performed. Voluntary muscular contraction and electrical neuromuscular stimulation were indicated and could be started on patient discharge. Patients were non-weight bearing for four weeks post-operatively, and a knee brace was used for support and knee protection. During the second month, the patients were allowed to move gradually toward full weight bearing, usually reached at eight weeks. Active functional training was then gradually started, with the goal of returning to a correct running pathway by proprioceptive muscular strengthening, endurance exercises, and aerobic training.

### Follow-up evaluation

Patients were evaluated pre-operatively and prospectively at 24 and 84 months using the Cartilage Standard Evaluation Form as proposed by the ICRS; in particular, the International Knee Documentation Committee (IKDC) subjective and IKDC objective scores were adopted. The lowest ratings in effusion, passive motion deficit, and ligament examination were used to determine the final functional grade of the knee (A—normal, B—nearly normal, C—abnormal or D—severely abnormal). The sport level was evaluated with the Tegner activity score. Adverse events and failures were also recorded. The operation was deemed to have failed if the patient needed a re-operation because of symptoms due to the primary defect. For failed patients, the last clinical assessment before re-operation was considered for final evaluation. Besides surgical failures, patients without a clinically significant improvement (10 IKDC subj points as per literature definition [14]) with respect to the basal evaluation were considered clinical failures.

### Statistical analysis

All continuous data were expressed in terms of mean ± SD; categorical variables were expressed as proportions or percentages. The Shapiro-Wilk test was performed to test normality of continuous variables. Repeated-measures GLM with post hoc Sidak correction for multiple comparisons was performed to compare the scores at different follow-up times. The ANOVA test was performed to assess the between-group differences of continuous normally distributed and homoscedastic data; the Mann Whitney test was used otherwise. The ANOVA test followed by the Scheffè post hoc pairwise comparison was used also to assess the among-group differences of continuous, normally distributed and homoscedastic data; the Kruskal Wallis test followed by the Mann Whitney test with the Bonferroni correction for multiple comparison was used otherwise. The Spearman rank correlation was used to assess correlations between scores and continuous data. Fisher’s exact test was performed to investigate relationships between grouping variables. The Kaplan-Meier survival analysis was performed to check the survival to failure, the log-rank test was used to assess the influence of categorical factors to the survival, and the Cox regression was used to assess the influence of continuous factors for survival. For all tests, *p* < 0.05 was considered significant. All statistical analysis was performed using SPSS v.19.0 (IBM Corp., Armonk, NY, USA).

## Results

A total of 27 consecutive patients affected by OCD lesions of the femoral condyles were included in the current analysis, according to inclusion/exclusion criteria. Fifteen patients were treated with OAT, whereas 12 patients were treated with PG (detailed characteristic of the included patients are reported in Table 1). All procedures were performed over a time period of seven years from 2006 to 2013. The baseline characteristics were comparable among OAT and PG: mean age was 22 years (range 16–48) in OAT and 24 years (range 17–40) in PG; mean defect size was 2.2 cm^2^ (range 1–4.5) in OAT and 2.6 cm^2^ (range 1.5–4) in PG. More patients in the OAT group had previously undergone surgical procedures (7 patients: two microfracture (MFX) procedures, one PG, one cell-free osteochondral scaffold, one cartilage shrinkage, one undefined cartilage treatment, one arthrolysis) compared with the PG group (4 patients: three fragment excisions, one synovial plica excision, one MFX procedure, one meniscectomy, one LCA reconstruction), although this difference was not statistically significant. As per inclusion criteria, no patient underwent combined procedures. The only significant difference between the two groups regarded the surgical approach, with more patients undergoing an arthroscopic approach in the PG group.

**Table 1:** Characteristics of OAT and PG patients. *OAT* osteochondral autologous transplantation, *PG* paste grafting technique, *M* male, *F* female, *MFC* medial femoral condyle, *LFC* lateral femoral condyle

	OAT	PG	Comparison
Age at time of surgery (year)	22.4 ± 7.2	24.2 ± 8.5	n.s.
Sex (M/F)	11/4	9/3	n.s.
Lesion site (MFC/LFC)	13/2	8/4	n.s.
Size (cm^2^)	2.2 ± 1	2.6 ± 1	n.s.
Previous surgery	46.6%	33.3%	n.s.
Previous cartilage surgery	40%	8.3%	n.s.
Approach (open/arthroscopic)	11/4	1/11	*p* = 0.001

**Fig. 2:**
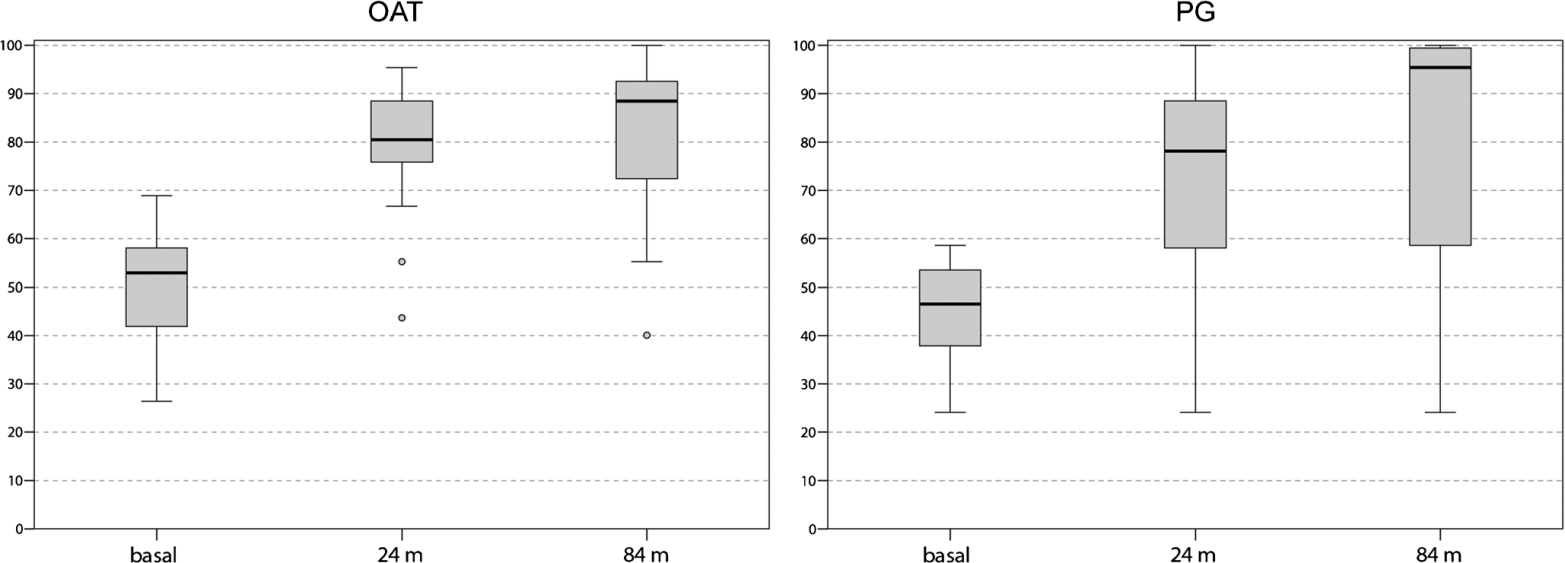
IKDC subjective scores before surgery, at 24 months after surgery, and at the final follow-up at 84 months after surgery, for OAT and PG. OAT: baseline–24 months *p* < 0.0005; baseline–84 months *p* < 0.0005; 24–84 months *p* = 0.044; PG: baseline–24 months *p* = 0.008; baseline–84 months *p* = 0.005; 24–84 months *p* = 0.025; OAT vs PG: baseline *p* = 0.054; 24 months *p* = 0.492; 84 months *p* = 0.708

The IKDC subjective score improved significantly in both groups (Fig. 2). At 24 months of follow-up, a significant improvement from 53.4 ± 9.1 to 80.8 ± 12.9 (*p* = 0.005) was obtained in the OAT group and from 44.6 ± 11.0 to 71.4 ± 25.3 in the PG group (*p* = 0.008). A further significant increase was observed at 84 months in both groups, with a final score of 88.3 ± 10.3 (*p* = 0.044 vs 24 months) in OAT and 78.8 ± 29.6 (*p* = 0.025 vs 24 months) in PG. No significant differences were found between OAT and PG at both follow-ups.

The IKDC objective score in the OAT group improved from four normal knees at baseline (4A, 10B, 1D) to seven at 24 months (7A, 8B) and to ten at 84 months of follow-up (10A, 4B, 1C), but without reaching a significant difference between follow-ups (*p* = 0.08). A positive trend was recorded in the PG group, changing from one knee rated as normal at the basal evaluation (1A, 8B, 1C, 2D) to nine at 24 months (9A, 2B, 1D) and to ten at 84 months (10A, 1B, 1D) (*p* = 0.001). No significant differences were detected in the IKDC objective evaluation between OAT and PG at both follow-ups.

**Fig. 3:**
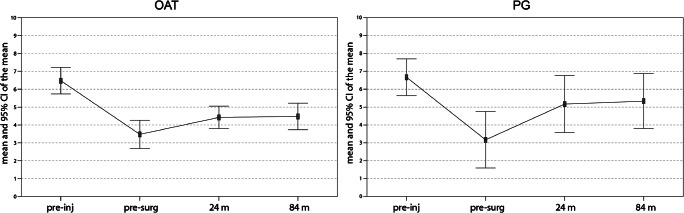
Tegner scores at pre-injury and pre-operative points, at 24 months after surgery, and at the final follow-up at 84 months after surgery, for OAT and PG. OAT: pre-surg–24 months *p* = 0.337; pre-surg–84 months *p* = 0.060; 24–84 months *p* = 0.289; PG: pre-surg–24 months *p* = 0.075; pre-surg–84 months *p* = 0.089; 24–84 months *p* = 0.995; OAT vs PG: pre-surg *p* = 0.074; 24 months *p* = 0.325; 84 months *p* = 0.221

The sport activity level, evaluated with the Tegner activity score, showed a tendency for improvement in both groups. Specifically, the level passed from a baseline level of 3.9 ± 1.4 and 3.2 ± 2.5 for OAT and PG, respectively, to 4.7 ± 0.9 and 5.2 ± 2.5 at 24 months and to 5.1 ± 0.9 and 5.3 ± 2.4 at 84 months (*p* = 0.060 and *p* = 0.089 for OAT and PG, respectively, between baseline and 84-months follow-up), without reaching the pre-injury level and with no significant differences between groups (Fig. 3).

Further analysis was performed to identify factors influencing the clinical outcome: sex, site and area of the lesion, history of previous surgical procedures or surgical approach did not influence the results in this series.

**Fig. 4:**
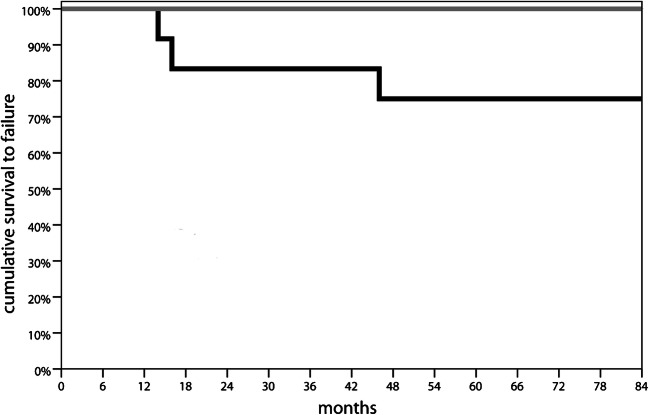
Survival curve. Grey line: OAT; Black line: PG

Regarding adverse events, one patient of the OAT group required knee mobilization under narcosis two months after surgery, due to articular stiffness. Two other patients within the OAT group complained for mid-term donor site morbidity with symptoms related to the graft harvest site. None of these patients was considered failure according to the definition used. During the 84 months of follow-up, three patients within the PG group required reoperation because of symptoms due to the primary defect (one patient underwent OAT after 14 months; one patient progressed to knee arthroplasty after 16 months; one patient was treated with osteochondral allograft transplantation after 46 months) and were considered failures, for a failure rate of 25%. No failure was recorded among patients in the OAT group. The log-rank test comparison of the survival distributions at seven years underlined a significantly worse outcome for the PG procedure (*p* = 0.043) (Fig. 4).

## Discussion

The main finding of the present study is that both OAT and PG provided satisfactory clinical results at mid-term follow-up in patients affected by knee OCD lesions. A significant higher survival rate was reported in the OAT group.

The importance of restoring the damaged articular surface is well acknowledged, in order to avoid premature joint degenerative progression with a significant risk of anticipated OA [15]. This is particularly true in OCD, which commonly affects a young active population, making a proper treatment paramount. Along with patient age, skeletal maturity and lesion stability represent the main determinant for a correct management [16]. In fact, whereas a non-operative treatment with strenuous activity restriction may often lead to OCD lesion healing in young patients with open physis and stable lesions [17], surgery is generally required for unstable lesions and in patients who have reached skeletal maturity. The attempt of preserving the affected osteochondral unit with drilling techniques [18] or fragment fixation [19] should be the primary aim of the treatment. However, especially after skeletal maturity, patients may present with a loose body not suitable for refixation [16]. In these cases, to avoid the mere fragment excision and its detrimental consequences [7], several different procedures are available, including regenerative procedures.

The first cell-based regenerative technique developed is autologous chondrocyte implantation (ACI), further modified with its “sandwich technique” to concomitantly address the subchondral bone, which was shown to provide durable results for OCD lesions leading to the regeneration of a hyaline-like tissue [20]. The field of regenerative treatments includes the ACI evolution into cell/scaffold-based matrix-assisted autologous chondrocyte transplantation (MACT), which can be combined with an autologous bone grafting in order to reconstruct both bone and cartilage, and provide articular surface restoration [21]. However, both techniques are limited by high costs and require two separate surgical procedures with demanding technical surgical issues, and inferior outcomes have been demonstrated in larger lesions [22]. The research for less expensive one-step procedures led to the development of biphasic cell-free osteochondral scaffolds, which were also applied in OCD lesions. Perdisa et al. [23] showed satisfactory clinical results at 60 months of follow-up, regardless of the defect size, suggesting the appropriateness of this procedure to treat also large OCD lesions. Nevertheless, MRI findings reported an incomplete bone regeneration, as reported also in other studies [24], demonstrating the difficulty to properly regenerate a physiologic subchondral bone tissue. To this regard, better results were proved for another procedure able of restoring the entire osteochondral unit in a single surgical procedure [22], the transplantation of fresh osteochondral allografts [25]. This demonstrated good clinical results also in knee OCD, but its main limitation is represented by graft availability and regulatory restrictions, with organization and distribution issues concurring to limit the possible application of this procedure to a few countries [22].

Thus, while many options have been documented, they are not always available, and OAT remains a broadly available option to reconstruct the articular surface. Nevertheless, OAT presents size limitations due to donor site morbidity, as lesions larger than 6 cm^2^ are associated with a poor prognosis, even when multiple graft plugs are used [26]. Regardless, with the proper indication, OAT still represents a valuable treatment also for OCD lesions. Smolders et al. [27] described satisfactory results treating OCD lesions ranging from 0.5 to 3.2 cm^2^, and other studies reported that OAT provides good to excellent results when applied for smaller articular cartilage between 1 and 4 cm^2^ [28]. However, even when applied with the proper indication, other OAT limitations remain due to the fibrotic tissue formation between plugs and the risk of articular cartilage anatomical incongruence between donor and recipient sites [29]. In this light, PG has been proposed to overcome OAT drawbacks, taking advantage from its intrinsic versatility, making it possible to treat also larger OCD lesions, independently of their location and shape, with minimal donor site morbidity [10]. The few available studies [11, 12] have shown promising findings on the reliability of PG as a treatment for OCD lesions at short- to mid-term follow-up. Nevertheless, very little is known about the comparison between OAT and PG, with small cohorts compared at different follow-ups without being able to show any difference [11].

In the present study, 15 patients treated with OAT and 12 with PG for OCD lesions of the femoral condyles were documented, demonstrating a significant clinical improvement up to seven years follow-up for both techniques, without any differences found between groups. In this survey, only relatively small OCD lesions up to 4.5 cm^2^ were treated, and the size of the lesions was not found to affect clinical outcomes in both OAT and PG group. The lack of large defects treated may also reflect on the low occurrence of procedure-related complications. In fact, in the current study, only 2 patients in the OAT group reported mild symptoms related to donor site at mid-term follow-up. This frequency is consistent with the results of a systematic review about donor site complaints associated with harvesting of osteochondral plugs from the knee joint, which reported a 5.9% occurrence of donor site morbidity for knee-to-knee mosaicplasty procedures [30]. A lower number of adverse events were documented instead for PG, which, on the other hand, presented a higher number of failures in this series.

Three patients within the PG group required further surgery as they experienced persistent pain and functional impairment, thus resulting in a relevant 25% failure rate, especially if compared with the better survival of OAT procedures, with no patients failing, according to both surgical and clinical definition. These rates are in line with previous studies, which reported a higher failure rate for PG with respect to OAT. In fact, Stone el al. [12] reported an even higher rate of re-operations, with five of the seven patients with OCD lesions treated with PG showing incomplete healing and requiring additional surgery. However, it should be considered that this study included only patients with a history of failed surgical treatments and thus may represent more complex cases. The literature also confirms the higher survival for OAT. In fact, no failures were demonstrated for OAT at a mean follow-up of 4.2 years in a comparative study versus MFX, which in turn presented a 41% failure rate [31]. Other techniques reported instead a higher survival, comparable with OAT, for the treatment of OCD lesions at mid-term follow-up. For example, Peterson et al. [32] reported only 3 failures among a survey of 58 patients treated with ACI for OCD knee lesions and evaluated at mean 5.6 years of follow-up. Similarly, Filardo et al. [21] reported four failures among 34 patients treated with MACT associated with bone grafting evaluated at six years follow-up, and all these failures occurred in larger lesions. The more recently introduced biphasic cell-free osteochondral scaffold provided encouraging findings to treat OCD lesions, with Perdisa et al. [23] reporting no failures at a five year follow-up in a survey of 27 patients. Finally, also fresh osteochondral allografts were found to provide a high mid-term survival, as shown by Cotter et al. [33], who followed 37 patients at an average of seven years of follow-up and reported a 5.1% failure rate, also highlighting a high rate of return to sport (81.8%) among athletes, at an average of 14.0 ± 8.7 months.

Sport activity represents indeed an important outcome to be considered in such young populations, and previous evidence about both OAT and PG reported satisfactory results. In particular, OAT was shown to provide a high rate of return to sport in patients affected by chondral and osteochondral lesions, with faster sport resumption compared with other techniques [34]. A recent meta-analysis demonstrated a 93% return to sport rate taking into account 261 patients treated with OAT for articular cartilage lesions of the knee. This result was higher with respect to osteochondral allograft, ACI, and MFX that showed an 88%, 82%, and 58% of return to sport rate, respectively. Also, PG demonstrated satisfactory results from a sport-activity point of view, with Stone et al. [12] reporting a significant Tegner score improvement at mid-term follow-up. The current analysis demonstrated only a tendency for improvement in sport activity for both PG and OAT, probably to the limited size of the surveys, and its design did not allow evaluating any differences about the time to return to sport between the two groups.

The main limitation of this study is represented by its retrospective design with consequent lack of randomisation, even though data were collected prospectively, and the strict inclusion/exclusion criteria for this analysis allowed comparing two groups which were similar for almost all variables. The only significant difference between the groups regarded the surgical approach, with PG mainly operated arthroscopically and OAT with a mini-arthrotomic approach. Nevertheless, no significant differences were found in the outcomes according to the approaches, and this is in line with previous literature findings. In fact, although it is true that the arthroscopic approach, resulting in reduced surgical trauma and mechanoreceptor disruption, reduces surgical morbidity and has an effect on rehabilitation and faster functional recovery, this was shown to affect only the short-term results, whereas it does not significantly influence the outcome beyond two years after surgery [35]. The absence of a radiological evaluation is another weak point, since MRIs would provide insights about graft integration and maturation. Regarding this topic, previous studies have already reported that PG was correlated with surface alteration in half of the patients, and with subchondral alteration in all of them [12], whereas OAT showed good mid-term MRI results [31], likely thanks to the transplantation of an already formed bone structure. Even more interesting would be a radiological evaluation to understand the effect of these surgical procedures on the natural history of joints affected by OCD in terms of OA development. Larger studies at longer follow-ups are needed, including also different surgical techniques to restore the articular surface and the osteochondral unit in patients affected by OCD, in order to understand the best surgical option to treat these young patients. More recently, other techniques based on autologous minced or particulated cartilage have been described [36–38] demonstrating the interest toward this autologous and low-cost treatment option. Nevertheless, while these approaches take advantage of the combination of biologics through the augmentation with other products such as fibrin glue, platelet-rich plasma, etc., only one study analysed the early results of eight patients affected by OCD lesions treated with autologous dual-tissue transplantation [38]. Thus, results are only preliminary and more data are needed to clearly demonstrate the benefit of one strategy versus the others to exploit the potential of minced or particulated cartilage. In this light, the current study sheds some light by comparing two procedures: based on the findings of the present study, both PG and OAT can be considered a valuable option to treat OCD lesions, with satisfactory results provided up to mid-term follow-up. However, while PG presents the advantages of a less invasive approach with lower adverse events, the higher failure rate should be considered when choosing among the treatment options to restore the articular surface in patients affected by knee OCD.

## Data Availability

Not applicable.
